# Tumor-like pulmonary sarcoidosis diagnosed by CT-guided transthoracic lung biopsy

**DOI:** 10.1186/1757-1626-2-6607

**Published:** 2009-05-15

**Authors:** N Sidiropoulou, P Filippousis, S Apostolopoulou, I Tsangaridou, L Thanos

**Affiliations:** “Sotiria”, Athens General Hospital of Chest Diseases, Department of Medical Imaging and Interventional RadiologyAthensGreece

## Abstract

**Introduction:**

Most patients referred for lung biopsy have a focal lesion that is likely to be a carcinoma and fine needle aspiration (FNA) is usually sufficient to confirm diagnosis. Percutaneous cutting needle biopsy (CNB) is an important and potential diagnostic technique when non carcinomatous disease is suspected or when the pulmonary disease is unclear, so tissue architecture is very important.

**Case presentation:**

We present a case of a 24 year old male arrived at our hospital with dyspnea and unusual computed tomography (CT) findings of sarcoidosis. Chest X-ray and CT scan revealed multiple masses in both lungs suggesting lung metastasis. Bronchoscopy and bronchoalveolar lavage did not reveal any malignant cells. None of the laboratory examinations revealed any primary extrapulmonary tumor. The patient underwent CT-guided core needle biopsy. Histopathological examination confirmed the diagnosis of sarcoidosis.

**Conclusion:**

CT-guided core needle biopsy is a very helpful diagnostic tool in order to determine the benign or malignant nature of a thoracic lesion.

## Introduction

FNA of focal pulmonary opacities is an accepted technique for the diagnosis of carcinoma, with a reported sensitivity greater than 90% [1-2].

In non-carcinomatous masses, its sensitivity is 22-48% [1-4].

Complication rates are of 29-45% [3].

However it is much less successful in the diagnosis of benign focal lesions, diffuse lung disease (DLD), or when the pulmonary disease is unclear, in which case tissue architecture is important [2-5].

CNB on the other side, provides cores of tissue for histological evaluation, although the American Thoracic Society Guidelines have stated that CNB should not be used in the diagnosis of DLD [6].

However it is an increasing safe procedure in the determination of malignant and non-malignant lesions.

The alternatives to percutaneous biopsy include open biopsy, video-assisted thoracoscopic biopsy and transbronchial biopsy. All of these however require general anesthesia.

## Case presentation

A 24-year old male (of greek origin and nationality) presented to our hospital complaining dyspnea.

He underwent a chest X-ray that revealed multiple bilateral pulmonary masses.

**Figure 1.**
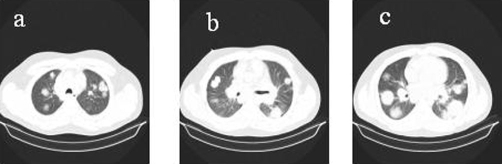
Non contrast chest CT scan, pulmonary window: multiple bilateral masses, in a patchy distribution, of increased density and lobulated contour. Some have a ground-glass opacity. Associated mediastinal lymphadenopathy (bilateral hilar, subcarinal, paratracheal) is noted, which is better appreciated using this window.

**Figure 2.**
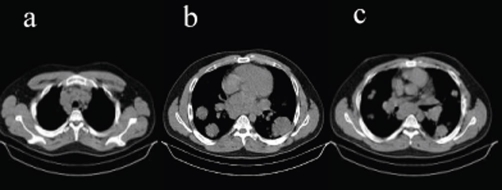
Non contrast chest CT scan, mediastinal window: multiple, bilateral, heterogeneous masses, with lobulated margins and mediastinal lymphadenopathy are noted.

The patient underwent a chest CT scan (Siemens, Somatom Emotion Duo, spiral tomography, 5 mm slice thickness, mediastinal and pulmonary window), that revealed well circumscribed multiple round masses, involving both lungs, suggesting lung metastasis and enlarged mediastinal lymph nodes (fig. 1,2).

Further imaging exams were performed (abdominal CT scan), which excluded any primary extrapulmonary tumor. The patient underwent other laboratory exams (blood tests) which had negative results.

The patient therefore underwent a CT-guided core biopsy using a 18 gauge (G) cutting needle (Somatex, medical technologies, GmbH, 18 G, 100 mm length).

The method was performed under local anesthesia.

The patient was placed in the appropriate position considering the location of the lesion.

Using a spiral CT, 5 mm slices were taken in order to specify the exact skin entry point, for the needle insertion.

The puncture point was prepared in a sterile fashion and then a 20 G needle was inserted.

**Figure 3.**
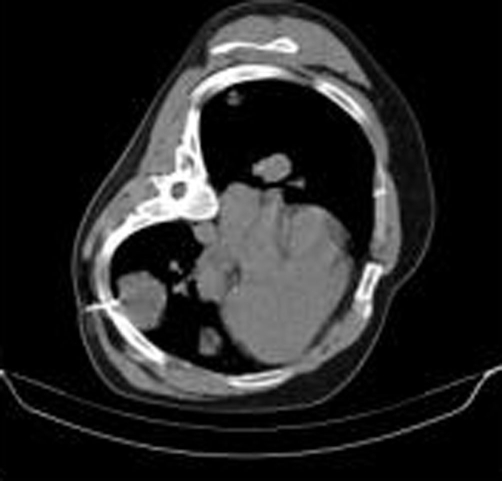
Chest non-enhancing CT scan, mediastinal window: the cutting needle was inserted in the chosen point. Patient was placed in left decubitus position in order to achieve the right pathway to the chosen lesion to be biopsied. The tip of the needle is seen inside the lesion.

A few CT images were taken to ensure that the chosen point of entry was the appropriate. Then, local anesthetic (20 cc) 2% lydocaine (xylocaine) was instilled. After that, a 18 G and 10 mm long cutting needle was inserted (fig. 3).

The distance between the skin and the edge of the lesion was calculated from a relevant CT image. 1-3 specimens were prelevated from the lesion, which were then put in formalin for histological examination.

The procedure was successful.

No complications occurred.

Histopathological examination confirmed the diagnosis of stage II sarcoidosis. It revealed multiple epitelioid cell granulomas with giant cells, in the absence of microbiological or virological abnormalities.

## Discussion

The first lung core-needle biopsies were reported by Dutra and Geraci in 1954 [7] after Silverman introduced the cutting needle in 1938.

The procedure enjoyed an early period of success in the diagnosis of focal malignant [7-8], focal benign [8] and diffuse pulmonary disease [9].

However, series of reported fatalities during the 70's mainly due to hemorrhage and pneumothorax rates, compared to FNA, cast it into disfavor [10-11].

In 1989 the American Thoracic Society Guidelines stated that CNB remained much less sensitive than open biopsy in the diagnosis of DLD with a sensitivity of 53-94%.

The automatic cutting needle biopsy devices introduced over the last few decades, appear to be safer than the older needles.

Complication rates in recent series are 15-33%.

Mortality rates are 0% [1,3,12,13].

Specificity of the CNB is 100%.

Sarcoidosis is a disease of unknown etiology characterized by non-necrotizing granulomatous inflammation involving one or more organs.

Estimates vary, but between 20 to 40% of patients present asymptomatic bilateral hilar lymphadenopathy [14]. In the remainder, a variety of tissues may be affected and mass lesions may result.

Tests formerly believed to be diagnostic of sarcoidosis, have proven to be non-specific.

These include serum ACE, which can be elevated in a variety of granulomatous diseases and BAL fluid analysis [15].

The Kwiem-Stilzbach skin test, although highly specific, is not standardized and is not widely available.

In patients who present findings uncharacteristic of sarcoidosis, such as isolated superficial and/or deep parenchymal nodules or masses and no previous clinical history that suggest the diagnosis, tissue diagnosis is necessary.

**Table.** Cardinal HRCT Features of Pulmonary Sarcoidosis

**Classical features (potentially reversible)**
• Intrathoracic lymphadenopathy (hilar, mediastinal, para-aortic, subcarinal)
• Proclivity for upper and mid-lung zones; perihilar (axial) distribution
• Lesions follow bronchovascular bundles, lymphatics
• Nodules (microscopic; macroscopic)
• Consolidation (mass-like lesions; confluent alveolar opacities)
• Ground glass opacities
• Thickened bronchovascular bundles; interlobular septa
• Non septal lines; subpleural linear opacities
**Chronic findings (reflect irreversible fibrosis)**
• Bronchiectasis; bronchiolectasis
• Cysts; bullae; paracicatricial emphysema
• Anatomic distortion (fissure, bronchi, vessel, displacement)
• Volume loss (particularly upper lobes; hilar retraction)
• Mycetoma (within pre-existing cavity)
• Calcified lymph nodes

## Conclusion

Core needle biopsy under CT guidance, is a very helpful, minimally invasive technique in the determination of malignant and non malignant lesions, with a few associated complications.

We showed successfully the usefulness of the method in the diagnosis of sarcoidosis with unusual CT scan findings.

## List of abbreviations

ACE: angiotensin converting enzyme
BAL: bronchoalveolar lavage
CNB: core needle biopsy
CT: computed tomography
DLD: diffuse lung disease
FNA: fine needle biopsy
G: gauge
HRCT: high resolution computed tomography
